# Assessment of Driving Status and Notification of DVLA Advice Within an Acute Inpatient Psychiatric Setting – a Closed Loop Audit

**DOI:** 10.1192/bjo.2026.11686

**Published:** 2026-06-30

**Authors:** Uwaila Olotu, Siyament Sacaklidir, George Jones, Francis Donya, Sumeyo Ahmed

**Affiliations:** Surrey and Borders Partnership NHS Trust, Guildford, United Kingdom

## Abstract

**Aims:**

Current DVLA guidelines advice that patients admitted to hospital for serious mental health conditions should not drive for 3 months following stabilisation and should inform the DVLA of their mental status. This closed loop audit, completed on a Working-Age Adult ward, evaluated current practice and introduced an intervention with subsequent re-evaluation.

**Methods:**

A baseline audit was conducted (n=12) which demonstrated that driving status was explored 25% of the time, and of these 67% were given DVLA advice and 67% were told to inform the DVLA. It was concluded that driving status was not routinely involved in MDT discussions, with a clear scope of change. Therefore, an intervention was introduced – the addition of a “driving status” to the electronic ward round template to standardise screening during ward reviews. A prospective re-audit during a 10-week period of 20 patients was then completed. The aims were to implement a sustainable change to improve on current practice and to re-audit to assess impact of intervention. Measured parameters were driving status exploration, advice given regarding driving and was advice to contact DVLA provided.

**Results:**

Following re-auditing, driving status exploration had improved to 85%.Furthermore, of that cohort that drove, 86% were given the relevant DVLA advice regarding whether they could drive or not, an increase from 67% at baseline. Of that cohort that drove 71% were advised to contact the DVLA, as opposed to 67% at baseline. These results suggest that the electronic ward round template intervention introduced did significantly improve the exploration of driving status, and that in this cohort the providing of relevant DVLA advice was improved.

**Conclusion:**

Some of the strengths of the audit included its closed-loop design, which allowed for an evaluation of the intervention introduced. There was also an increase in sample size from the baseline audit to the re-audit. Additionally, the simplicity of the intervention allowed for a sustainable change in practice. Limitations of the audit included the sample size, which reflected a single ward in the hospital and as a result may not generalise across other services. Further actions included introductions of posters on the ward, qualitative information gathering of MDT awareness and expansion of audit into other wards in the hospital.

